# Antibacterial Activities of Prenylated Isoflavones from *Maclura tricuspidata* against Fish Pathogenic *Streptococcus*: Their Structure-Activity Relationships and Extraction Optimization

**DOI:** 10.3390/molecules26247451

**Published:** 2021-12-09

**Authors:** Jae-Woong Lim, Yang Hee Jo, Ji-Seok Choi, Mi Kyeong Lee, Ki Yong Lee, So Young Kang

**Affiliations:** 1 Department of Aqualife Medicine, Chonnam National University, Yeosu 59626, Korea; yowoo98@gmail.com (J.-W.L.); clugy9607@naver.com (J.-S.C.); 2 College of Pharmacy, Chungbuk National University, Cheongju 28160, Korea; qow0125@naver.com (Y.H.J.); mklee@chungbuk.ac.kr (M.K.L.); 3 College of Pharmacy, Korea University, Sejong 30019, Korea; kylee11@korea.ac.kr

**Keywords:** *Maclura tricuspidata*, prenylated isoflavone, *Streptococcus*, principal component analysis, 6,8-diprenlygenistein

## Abstract

*Streptococcus* zoonotic bacteria cause serious problems in aquaculture with clinical effects on humans. A structure-antibacterial activity relationships analysis of 22 isoflavones isolated from *M. tricuspidata* (leaves, ripe fruits, and unripe fruits) against *S. iniae* revealed that prenylation of the isoflavone skeleton was an important key for their antibacterial activities (minimum inhibitory concentrations: 1.95–500 μg/mL). Through principal component analysis, characteristic prenylated isoflavones such as 6,8-diprenlygenistein (**4**) were identified as pivotal compounds that largely determine each part’s antibacterial activities. *M. tiricuspidata* ripe fruits (MTF), which showed the highest antibacterial activity among the parts tested, were optimized for high antibacterial activity and low cytotoxicity on fathead minnow cells using Box–Behnken design. Optimized extraction conditions were deduced to be 50%/80 °C/7.5 h for ethanol concentration/extraction temperature/time, and OE-MTF showed contents of 6,8-diprenlygenistein (**4**), 2.09% with a MIC of 40 µg/mL. These results suggest that OE-MTF and its active isoflavones have promising potential as eco-friendly antibacterial agents against streptococcosis in aquaculture.

## 1. Introduction

Global fish production peaked at about 179 million tons in 2018, with aquaculture representing 46% of total fish production. Of produced fish, 52% were used for human consumption (excluding non-food uses). The total first sale value of aquaculture production in 2018 was estimated at USD 250 billion [1]. However, disease outbreaks are considered to be a significant constraint to the development of the aquaculture sector. Economic losses due to diseases globally have been estimated to be in the range of several billion US dollars per year [2]. In Korea, the olive flounder (*Paralichthys olivaceus*) is one of the most commercially important marine flatfish species for aquaculture. The production of olive flounder was 43,320 tons in 2019, which was 50.8% of the total production output, the highest proportion for a single fish aquaculture species in Korea (Korean Statistical Information Service, KOSIS, http://kosis.kr, accessed on 24 February 2021).

However, infectious bacterial diseases such as streptococcosis, vibriosis, and edwardsiellosis are major problems for olive flounder aquaculture in Korea [3]. Streptococcosis caused by bacteria in the genus *Streptococcus* is one of the major causes of mortality of farmed olive flounder in Korea [4]. Additionally, *Streptococcus* is an important zoonotic bacteria. It not only causes serious problems in aquaculture, but also has clinical effects on humans. In humans, *Streptococcus iniae* can cause infection via an external wound or ulcer. It can colonize the nasal mucosa and then become systemic, causing endocarditis, cellulitis, meningitis, and systemic arthritis [5,6]. Therefore, controlling this pathogen in aquaculture is also important for public health.

As a measure against such bacterial diseases, antibiotics such as amoxicillin, florfenicol, and oxytetracycline have been used to minimize economic losses throughout South Korea for decades [3]. However, the frequent use of antibiotics has serious drawbacks, such as increased antibiotic resistance, environmental contamination, toxicity to the host, and contamination of fish products with drug residues [7,8]. Therefore, anti-streptococcosis strategies using safe and effective alternative agents are urgently needed to solve these problems. Many studies have indicated that natural products have potentials as effective immunostimulating and anti-pathogenic agents for cultured fish [2,9,10].

*Maclura tricuspidata* (Moraceae, also known as *Cudrania tricuspidata* (carr.) Bur.) is a thorny tree cultivated in East Asia, including Korea. Its edible fruit has been made into juices, jams, alcoholic beverages, dietary supplements, and other health products in Korea [11,12]. In addition, the stems and roots of *M. tricuspidata* have been used as herbal teas or functional beverages in China [12]. The leaves of *M. tricuspidata* have been mainly used for growing silkworms or as an herbal tea in Korea [13].

Phytochemicals (such as anthocyanin, flavonoids, and polyphenols) present in the leaves and fruit of *M. tricuspidata* are known to have biological functions, such as antibacterial, anti-obesity, antioxidant, anti-inflammatory, and immunomodulatory activities [14,15,16]. Recently, it has been reported that prenylated isoflavonoids are major compounds in *M. tricuspidata* [11,14,17]. These prenylated isoflavonoids have strong antibacterial activities against human pathogenic Gram-positive bacteria such as *Listeria monocytogenes* and methicillin-resistant *Staphylococcus aureus* (MRSA) [18,19]. However, to the best of our knowledge, studies on the antibacterial activities of *M. tricuspidata* extracts and its active compounds against pathogenic bacteria in fish have not been reported yet. Therefore, in the present study, we investigated the antibacterial activity of isoflavones isolated from the leaves, ripe fruits, and unripe fruits of *M. tricuspidata* against fish pathogenic *Streptococcus* and their structure–activity relationships (SAR).

Generally, different parts of plants contain different components with diverse biological activities [20]. In particular, the maturation of fruits is one of the main factors that influences the compositions and contents of active components [21]. To select the optimal parts of *M. tricuspidata*, the antibacterial activities of various parts of *M. tricuspidata*, such as the leaves, ripe fruits, and unripe fruits, were compared. Differences amongst parts were evaluated and key antibacterial compounds in *M. tricuspidata* extracts were identified using liquid chromatography-quadrupole-time of flight mass spectrometry (LC-Q-TOF MS) and principal component analysis (PCA).

To develop anti-infection agents from effective natural products, an extraction procedure is indispensable. The extraction efficiency and biological activity of extracts are influenced by multiple parameters, such as the extraction temperature, extraction time, organic solvent composition, and solvent-to-sample ratio, independently or interactively [14,22,23,24]. To obtain effective and non-toxic antibacterial extracts from *M. tricuspidata* ripe fruits, extraction conditions such as ethanol concentration, extraction temperature, and extraction time were optimized to achieve high antibacterial activities against *S. iniae* while also achieving low cytotoxicity to fathead minnow cells using a three-factor–three-level Box–Behnken design (BBD). 

## 2. Materials and Methods

### 2.1. Materials and Reagents

For LC-Q-TOF MS analysis, high-performance liquid chromatography (HPLC)-grade water, methanol, acetonitrile, and formic acid were purchased from Honeywell B & J (Morristown, NJ, USA) and Thermo Fisher Scientific (Waltham, MA, USA). Amoxicillin, oxytetracycline and cell culture-grade dimethyl sulfoxide (DMSO) were purchased from Sigma (St. Louis, MO, USA). Brain heart infusion agar (BHIA) and broth (BHIB) were bought from Difco (Sparks, MD, USA).

### 2.2. Plant Materials

Dried leaves of *M. tricuspidata* (MTL) were purchased from a local herbal market in Chungbuk, Korea in October 2013. They were identified by the herbarium of the College of Pharmacy at Chungbuk National University, where a voucher specimen was deposited (CBNU201310-MTL). Fresh unripe fruits (MTU) and fresh ripe fruits (MTF) of *M. tricuspidata* were purchased from a local herbal market in Hampyeong-gun, Korea in May 2015 and October 2015, respectively. Their voucher specimens (CBNU201505-CTUF and CBNU201510-CTRF) were deposited at the herbarium of the College of Pharmacy, Chungbuk National University.

### 2.3. Extraction, Isolation, and Identification of Isoflavones from M. tricuspidata

Twenty-two isoflavones used in the present study were isolated and identified by analyses of spectroscopic data such as ultraviolet (UV, Jasco UV-550, Tokyo, Japan), ^1^H-nuclear magnetic resonance (^1^H-NMR, Bruker ADVANCE 400 or 500 MHz NMR spectrometer, Billerica, MA, USA) and MS (Thermo scientific LCQ Fleet ion trap MS, San Jose, CA, USA) spectra and comparisons with literature values as described in previous studies [11,17]. Their separation and identification methods are briefly described as follows (for detailed separation and identification methods, refer to Supplementary Materials: 1. Extraction, isolation, and identification of isoflavones from *M. tricuspidata*). Dried leaves of *M. tricuspidata* (0.8 kg) were extracted with 100% methanol (MeOH) twice, yielding a methanolic extract (102.4 g). This methanolic extract was then suspended in H_2_O and partitioned successively with *n*-hexane (12.2 g), dichloromethane (DCM) (15.2 g), ethyl acetate (EtOAc) (4.7 g), and *n*-butanol (*n*-BuOH) (17.7 g). The DCM fraction (15.2 g) was subjected to silica gel column chromatography (NP-MPLC) with a mixture of DCM and MeOH with increasing polarity to give 10 fractions (MTLM1–MTLM10). Fractions MTLM2, MTLM3, and MTLM4 were subjected to Sephadex LH-20 and semi-preparative HPLC to give compounds **3**, **4**, **5**, **6**, **7**, **8**, **9**, **10**, **11**, **15**, **17**, **18**, **19**, **20**, **21**, and **22**. The EtOAc fraction (4.7 g) was subjected to NP-MPLC with a mixture of DCM and MeOH with increasing polarity to give 9 fractions (MTLE1–MTLE9). Fractions MTLE3 and MTLE5 were subjected to column chromatography over Sephadex LH-20 and semi-preparative HPLC to give compounds **1** and **2**.

Fresh unripe fruits of *M. tricuspidata* (2.8 kg) were extracted successively with 75% ethanol at room temperature. The ethanolic extract (508.2 g) was then suspended in H_2_O and partitioned successively with *n*-hexane (30.1 g), DCM (44.6 g), EtOAc (7.5 g), and *n*-BuOH (35.8 g). The DCM fraction (44.6 g) was subjected to NP-MPLC with a gradient elution using *n*-hexane:EtOAc (50:1 ~ 0:100) to obtain 11 fractions (MTUM1–MTUM11). Fractions MTUM1 and MTUM6 to M11 were subjected to defatting, NP-MPLC, Sephadex LH-20, semi-preparative HPLC, and recrystallization to give compounds **2**, **3**, **5**, **6**, **7**, **10**, **11**, **12**, **13**, **14**, **15**, **16**, **17,** and **20**.

Fresh ripe fruits of *M. tricuspidata* (1.2 kg) were extracted twice with 100% MeOH, which yielded a methanolic extract (486.5 g). This methanolic extract was suspended in H_2_O and partitioned successively with *n*-hexane (8.8 g), DCM (14.4 g), EtOAc (4.3 g), and *n*-BuOH (19.5 g). The DCM fraction (14.4 g) was subjected to NP-MPLC and gradient elution with *n*-hexane:EtOAc (20:1 ~ 0:100) and EtOAc:MeOH (100:0 ~ 0:100) to give 11 subfractions (MTFM1–MTFM11). Subfractions MTFM4, MTFM7, and MTFM10 were subjected to reverse phase column chromatography (RP-MPLC), Sephadex LH-20, semi-preparative HPLC, and recrystallization to give compounds **1**, **2**, **3**, **4**, **5**, **8**, and **11**.

**Figure 1:**
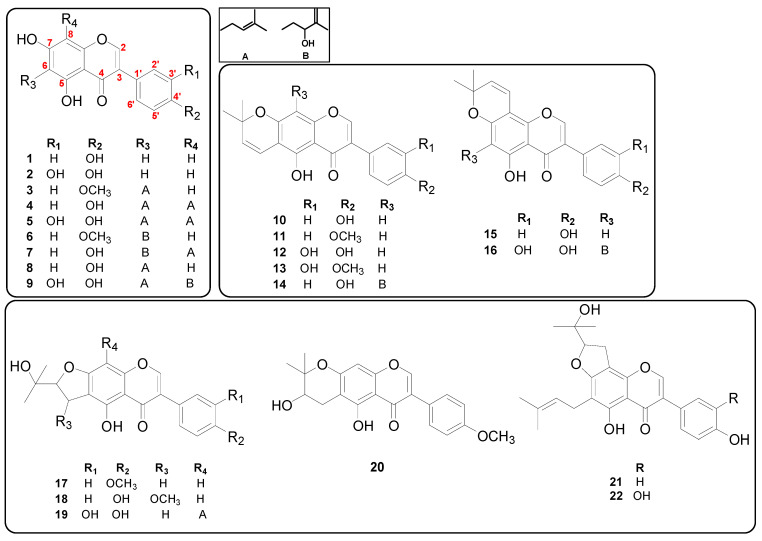
Chemical structures of 22 isoflavones tested for antibacterial activities in the present study.

A total of 22 isoflavones were identified as genistein (**1**), orobol (**2**), gancaonin A (**3**), 6,8-diprenylgenistein (**4**), 6,8-diprenylorobol (**5**), 5,7-dihydroxy-6-(2″-hydroxy-3″-methylbut-3″-enyl)-4′-methoxylisoflavone (**6**), isoerysenegalensein E (**7**), wighteone (**8**), millewanin H (**9**), alpinumisoflavone (**10**), 4′-*O*-methylalpinumisoflavone (**11**), 5,3′,4′-trihydroxy-6″,6″-dimethylpyrano-[2″,3″:7,6]isoflavone (**12**), 3′-hydroxy-4′-*O*-methylalpinumisoflavone (**13**), euchrenone b_8_ (**14**), derrone (**15**), 5, 3′,4′, 2‴-tetrahydroxy-2″, 2″-dimethylpyrano-(5″,6″:7,8)-6-(3‴-methyl-3‴-butenyl)isoflavone (**16**), 4′-*O*-methylerythrinin C (**17**), (±)-1″-*O*-methylerythrinin F (**18**), furowanin A (**19**), 4′-*O*-methyl-2″-hydroxydihydroalpinumisoflavone (**20**), senegalensin (**21**), and furowanin B (**22**) based on previous studies [11,17]. The chemical structures of these 22 isolated isoflavones are shown in Figure 1.

### 2.4. LC-Q-TOF MS Analysis of Isoflavones in Extracts of M. tricuspidata Leaves, Ripe Fruits, and Unripe Fruits with Principal Component Analyses (PCA)

Analysis of isoflavones in extracts was performed with an AB Sciex (Framingham, MA, USA) ExionLC coupled to an X500R Q-TOF mass spectrometer equipped with an electrospray ionization (ESI) ion source. Each extract (methanolic extracts from each part, 1 mg/mL in methanol, 10 µL) was injected into a Kinetex C_18_ column (150 × 4.6 mm, 5 µm; Phenomenex, CA, USA) connected to a short pre-column. The column was operated at 40 °C. The mobile phase consisted of 0.1% formic acid water solution (A) and acetonitrile (B). The following elution gradient was used: 0 to 30 min, linear gradient from 5 to 100% B; 30 to 40 min isocratic at 100% B; 40 to 40.1 min, linear gradient from 100 to 5% B; 40.1 to 50 min, isocratic at 5% B. The optimized LC-Q-TOF MS conditions were as follows: curtain gas, 25 psi.; ion source gas 1 and gas 2, 50 psi; gas temperature, 400 °C; ion spray voltage, 5500 V; declustering potential, 80 V; and flow rate, 1 mL/min (using 1/5 splitter). Information-dependent acquisition (IDA) mode was used to automatically trigger the MS/MS spectra acquisition when a chromatographic signal exceeded a threshold of 100 counts per second. Mass spectra were acquired in the *m*/*z* range of 100 to 1000. Molecular masses of precursor ions were accurately detected using reference masses.

PCA was performed to differentiate extracts according to semi-quantitative data of identified isoflavones. This allowed identification of isoflavones that most significantly affected each part’s extract. PC scores were auto-scaled. A MarkerView software (AB Sciex, CA, USA) was used for analysis.

### 2.5. Bacteria and Culture Conditions

*Streptococcus iniae* KCTC3657, *S. parauberis* KCTC3651, *Edwardsiella tarda* KCTC12267, and *Aeromonas salmonicida* KCCM40239 were purchased from Korean Collection for Type Cultures (Daejeon, Korea) and Korean Culture Center of Microorganisms (Seoul, Korea). Clinical strains *S. iniae* DSJ19 (Jeju island in Republic of Korea, 1997; used to test the antibacterial activity of 15 extracts from BBD), *S. parauberis* KSP44 (Jeju island in Republic of Korea, 1999), and *S. iniae* BS9 (Tongyoung in Republic of Korea, 1998) were originally isolated from a diseased olive flounder and identified by 16s rRNA gene sequencing. After subculturing in brain heart infusion broth (BHIB) for cryopreservation, aliquots of all bacterial strains were kept frozen at −80 °C in BHIB containing 14% glycerol until use.

### 2.6. Antibacterial Susceptibility Test

Antibacterial activity was evaluated with a broth dilution method (approved guideline: M49-A) as described by the Clinical and Laboratory Standards Institute [25] with slight modifications [26,27]. Briefly, bacterial colonies taken directly from brain heart infusion agar (BHIA) plates were incubated into BHIB and cultured at 25 °C for 24 h. From this culture, a suspension equivalent to 0.5 McFarland standard in BHIB was prepared. Isoflavones dissolved in BHIB (including 5% of DMSO (*v*/*v*) or less) and an equal volume of 1 × 10^6^ CFU/mL of bacteria were mixed in a 96-well plate and incubated at 25 °C for 24 h. Amoxicillin and oxytetracycline were used as reference controls. The lowest concentration of each antibiotic that visibly inhibited bacterial growth was considered the minimum inhibitory concentration (MIC). The minimum bactericidal concentration (MBC) was also determined. Briefly, the bacterial suspension at or above the MIC (20 µL of each well) was inoculated into a fresh broth (200 µL) and incubated for 25 °C for 24 h more. The lowest concentration where no growth was visually observed was considered the MBC. In the case of MBC/MIC ratio ≤ 4, the effect was considered to be bactericidal [28]. Each assay was repeated three times.

### 2.7. Time-Growth Curve and Scanning Electron Microscope (SEM) Analyses

Time-growth curve and SEM analyses were performed to investigate effects on bacteria of optimized extract from MTF (OE-MTF) against *S. iniae* DSJ19. Time-growth curves were made by the same method as in Section 2.6. Antibacterial susceptibility test. Absorbance was measured at 600 nm at 2 h intervals using the kinetic mode of a VERSA max microplate reader (Molecular Devices, CA, USA) at 25 °C. Amoxicillin, used as a reference control, was also tested. The same volume of BHIB (without any antibiotic and bacteria) was set as the blank.

The effect of OE-MTF on *S. iniae* was investigated by SEM following the method by Yun et al. [29]. with slight modifications. Briefly, 40 mL of *S. iniae* suspension (1 × 10^7^ CFU/mL) was mixed with OE-MTF at MIC of 40 μg/mL for 20 h. The control was prepared by mixing equal volumes of bacterial suspension without OE-MTF. Following the incubation, the bacterial pellet was harvested by centrifugation at 3500× *g* for 10 min and thoroughly washed 3 times with phosphate-buffered saline (PBS) to eliminate the residue of OE-MTF. Both treated and control bacteria pellets were treated in 2.5% glutaraldehyde and 1% osmium tetroxide and then analyzed using a scanning electron microscope (JSM-7610F Plus, JEOL, Tokyo, Japan).

### 2.8. Cytotoxicity Assay

The fathead minnow (FHM) cell line (American Type Culture Collection No. CCL-42) was cultured in Dulbecco’s modified Eagle medium (DMEM, Gibco, NY, USA) supplemented with 10% fetal bovine serum (FBS, Gibco, NY, USA), 50 IU/mL of penicillin, and 50 µg/mL of streptomycin (Gibco, NY, USA). This cell line was maintained at 20 °C. FHM cells were plated into 96-well plates at a density of 10^5^ cells/well and cultured overnight. Cytotoxicity was evaluated with a neutral red (NR) uptake assay as described by Thompson [30] with slight modifications [31,32]. All samples were dissolved in DMSO and diluted in a medium to adjust the final concentration of DMSO to be 0.1% (*v*/*v*) or less. FHM cells (10^5^ cells/well, 96-well plates) were treated with a medium containing 2-fold serially diluted samples and incubated at 20 °C for 96 h. Then, an NR working solution (50 µg/mL neutral red dye, Sigma, MO, USA) was added to each well and incubated at 20 °C for 2 h. Plates were washed with phosphate-buffered saline (PBS) twice. A solution containing 1% acetic acid in 50% ethanol was then added to each well to extract the dye for 10 min. The absorbance of the colored solution was measured at 540 nm and 690 nm with a VERSA max microplate reader (Molecular Devices, CA, USA). The growth rate as an index of cytotoxicity was calculated by dividing the absorbance of test cells by the absorbance of corresponding control cells. The 50% cytotoxic concentration (CC_50_) was calculated using Microsoft Excel.

### 2.9. Box-Behnken Design (BBD)

Three-factor BBD was applied in this study to investigate individual and interactive effects of ethanol concentration (X_1_), extraction temperature (X_2_), and extraction time (X_3_) as variables on antibacterial activity against *S. iniae* DSJ19 and cytotoxicity to FHM cells. These studied factors, along with their experimental levels, are presented in Table S1 (tables and figures marked with S found in Supplementary Materials). The whole design consisted of 15 experimental points carried out in a random order. A total of 3 replicates at the center of the design were used to allow for the estimation of a pure error sum of squares. Based on experimental data, regression analysis was performed and fitted into an empirical second-order polynomial model:

Y = β_0_ + β_1_X_1_ + β_2_X_2_ + β_3_X_3_ + β_12_X_1_X_2_ + β_13_X_1_X_3_ + β_23_X_2_X_3_ + β_11_X_1_^2^ + β_22_X_2_^2^ + β_33_X_3_^2^ (1)

where Y was the predicted response; β_0_ was the interception; β_1_, β_2_, and β_3_ were linear coefficients of the ethanol concentration, extraction temperature, and solvent-to-sample ratio, respectively; β_12_, β_13_, and β_23_ were interaction coefficients of the ethanol concentration, extraction temperature, and solvent-to-sample ratio, respectively; and β_11_, β_22_, and β_33_ were squared coefficients of the ethanol concentration, extraction temperature, and solvent-to-sample ratio, respectively. Linear, interaction, and squared coefficients were determined by least square regression followed by analysis of variance (ANOVA) using Minitab 14 software (PA, USA). Statistical significance was considered at *p* < 0.05.

### 2.10. Quantification of 6,8-Diprenylgenistein *(**4**)* in OE-MTF and 15 Extracts from BBD

Stock solution for 6,8-diprenylgenistein (**4**) was prepared with HPLC methanol as the solvent. Working calibration solutions were prepared by successive serial dilution of the stock solution with methanol to yield final concentrations of 5, 10, 25, 50, 100, 250, and 400 ng/mL. OE-MTF, and 15 extracts from BBD (Table S1) were dissolved in 50% methanol to prepare stock solutions at 1 mg/mL. These stock solutions were diluted to make working solutions at 1, 2.5, and 5 µg/mL for OE-MTF and 10 µg/mL for 15 extracts from BBD. An Exion LC (AB Sciex, Framingham, MA, USA) coupled to an X500R Q-TOF mass spectrometer equipped with an ESI ion source was used for quantitative and qualitative analyses. Detailed LC and MS conditions are described in Supplementary Materials: 2. Quantification of 6,8-diprenlygenistein (**4**) in OE-MTF.

#### Method Validation

An external standard method was utilized for quantification. Linearity was studied by diluting stock solution to a series of at least five concentrations. Calibration plots were then constructed after triplicate analysis of the solution by plotting the mean integrated chromatographic peak area against the corresponding concentration. The limit of detection (LOD) and limit of quantification (LOQ) were calculated following International Conference on Harmonization (ICH) Q2B Guidelines.

The precision of the method was validated by determining intra- and inter-day variances. Intra-day precision was performed with three replications prepared from combined extract within one day. Inter-day precision was performed over three consecutive days. Relative standard deviation (RSD) was taken as a measure of precision.

Recovery was used to further evaluate the accuracy of the method. Known amounts of standard solutions were mixed with known amounts of samples. Resultant samples were then extracted and analyzed with the proposed method. Triplicate experiments were repeated at each level. Average recoveries were estimated with the following formula: recovery (%) = (amount found − original amount)/amount spiked × 100%; RSD (%) = (SD/mean) × 100%.

Specificity was achieved by analyzing multiple reaction monitoring (MRM) signals (Figure S3). All peaks of target compounds in the OE-MTF sample were identified by comparing retention time, parent ions, and product ions with standards in MRM spectra. All results related to quantitative analysis are included in Supplementary Materials: 2. Quantification of 6,8-diprenlygenistein (**4**) in OE-MTF.

### 2.11. Statistical Analysis

Analysis of variance (ANOVA) was used to identify the main effect, curvature effect, and interaction effect of major factors by extraction conditions using MINITAB release 14 (Minitab Inc., State College, PA, USA). Statistical significance was considered at *p* < 0.05 for all analyses.

## 3. Results and Discussion

### 3.1. Antibacterial Activities of Isoflavones from M. tricuspidata against S. iniae and Their Structure–Activity Relationships (SAR)

#### 3.1.1. Effects of Prenyl Group—Addition

The antibacterial activities of 22 isoflavones from *M. tricuspidata* were evaluated using *S. iniae* (Table 1). The MIC of compound **1**, an isoflavone without any prenyl substituent, was >500 μg/mL. However, compounds **3**, **4**, **5**, **6**, **7**, **8**, and **9** as prenylated isoflavones showed excellent antibacterial activities against *S. iniae*, with MIC values of 1.95 to 62.5 µg/mL. Among them, di-prenylated compounds **4**, **5**, **7**, and **9** had MICs of 1.95–15.63 µg/mL, showing much more enhanced antibacterial activities than mono-prenylated compounds **3**, **6**, and **8**. Cyclized compounds showed also similar results. Compound **10**, in which the prenyl group of C-6 was cyclized with the hydroxyl group of C-7, showed an MIC > 500 μg/mL while compound **14** had an MIC of 62.5 μg/mL. The only difference between these two compounds was that compound **14** had a hydroxy-prenyl group at C-8. Recent studies have also reported that amphiphilic features of isoflavone skeletons due to the addition of a prenyl group play an important role in their antibacterial properties [33], and that di-prenylated flavanones exhibit higher antibacterial activities than mono-prenylated ones [19,34].

#### 3.1.2. Effect of Cyclization of Prenyl Group

It was found that the cyclization of the prenyl group in prenylated isoflavones decreased their antibacterial activities against *S. iniae*. Compound **10**, which was cyclized between the hydroxyl at C-7 and the prenyl group at C-6 of the A-ring of compound **8** (MIC of 7.81 µg/mL), showed markedly reduced antibacterial activity with an MIC > 500 µg/mL. Similar results were observed for compound **11** (MIC of 250 µg/mL) vs. compound **3** (MIC of 62.5 µg/mL) and compound **20** (MIC of 125 µg/mL) vs. compound **6** (MIC of 31.25 µg/mL).

In addition, even for cyclized ones, the existence of a linear prenyl group at C-6 (compound **22**, MIC of 31.25 µg/mL) was more potent than the one with a linear prenyl group at C-8 (compound **19**, MIC of 62.5 µg/mL). Previous studies have also reported that the presence of a prenyl group at C-6 of the A-ring can improve antibacterial activities [18,33,35].

#### 3.1.3. Effects of Other Substituents

The hydroxyl group in the B-ring influenced the antibacterial activities of prenylated isoflavones against *S. iniae*. The presence of a di-hydroxyl group at B-ring as in compounds **2** and **12** (MIC: 500 and 31.25 µg/mL, respectively) resulted in a stronger antibacterial activity against *S. iniae* than the presence of a mono-hydroxyl group, as in compounds **1** and **10** (MIC > 500 µg/mL). The difference in antibacterial activity with respect to the number of the hydroxyl groups in the B-ring might be related to the affinity of these hydroxyl groups to proteins. According to Wang et al. [36], bacterial neuraminidase inhibitory activity is increased when the number of the hydroxyl groups of the B-ring in prenylated isoflavones is increased from 1 to 2. Dhayakaran et al. [37] have also reported that hydroxyl groups in isoflavones can promote enzyme inhibition and the inhibition of biosynthetic pathways in bacteria, as they have high affinities for proteins. However, the addition of a hydroxyl group to the B-ring tended to decrease the antibacterial activity. For example, compound **21** had an MIC of 7.81 µg/mL, whereas compound **22** with the addition of a hydroxyl group to the B-ring showed an MIC of 31.25 µg/mL. To clarify the effect of the number of hydroxyl groups on the antibacterial activities of prenylated isoflavones, further studies using a variety of derivatives are needed. 

With regard to *O*-methylation, methylation in prenylated isoflavones tended to decrease the antibacterial activity. Compound **3**, which had *O*-methylation at R_2_ in the B-ring of compound **8** (MIC of 7.81 µg/mL), showed a significantly reduced antibacterial activity, with an MIC of 62.5 µg/mL. The same trend was also found for compound **13** (*O*-methylated, MIC: 250 µg/mL) vs. compound **12** (MIC: 31.25 µg/mL).

### 3.2. Antibacterial Activities of Prenylated Isoflavones against Fish Pathogenic Clinical Strains of Streptococcus

The five most active prenylated isoflavones, **4**, **5**, **7**, **8**, and **21**, also showed excellent antibacterial activities against fish pathogenic clinical strains of *S. parauberis* and *S. iniae*, with MICs ranging from 1.95 to 31.25 µg/mL. In particular, di-prenylated isoflavones **4** (6,8-diprenylgenistein) and **7** (isoerysenegalensein E) exhibited bactericidal activities against *Streptococcus*, showing an MBC/MIC ratio ≤ 4. Furthermore, their bactericidal activities against *S. parauberis* were higher than those of amoxicillin, a clinically used antibiotic for streptococcosis in farmed fish (Table 2).

Many studies have reported on the excellent antibacterial activities of prenylated (iso)flavonoids against methicillin-resistant *Staphylococcus aureus* (MRSA). However, they lack absolute specificity in the mechanism of action of this class [33]. Generally, cytoplasmic membrane disruption is known to be the dominant mechanism of action of these (iso)flavonoids [19]. Because of their relatively high hydrophobicity, prenylated isoflavones are expected to show high affinity for the cytoplasmic membrane [19,38]. Based on this evidence, the excellent antibacterial activities of prenylated isoflavones against *Streptococcus* strains shown in the present study might be due to their high hydrophobicity.

### 3.3. Antibacterial Activities of Extracts from Each Part of M. tricuspidata against Fish Pathogenic Bacteria and Principal Component Analysis (PCA) Using LC-Q-TOF MS

To evaluate the field applications of extracts containing prenylated isoflavones having excellent antibacterial activities, we investigated their antibacterial activities against Gram-positive *S. iniae* and *S. parauberis* and Gram-negative *E. tarda* and *A. salmonicida*. Among MTL, MTF, and MTU extracts, MTF and MTL extracts only showed potent antibacterial activities against fish pathogenic Gram-positive *S. iniae* and *S. parauberis*, with MIC values of 62.5 to 1000 µg/mL. The MTF extract showed the highest antibacterial activities against fish pathogenic Gram-positive *S. iniae* and *S. parauberis* (MIC values of 62.5 and 250 µg/mL, respectively, Table 3). This suggests that it has a high potential to be used for further development.

**Figure 2:**
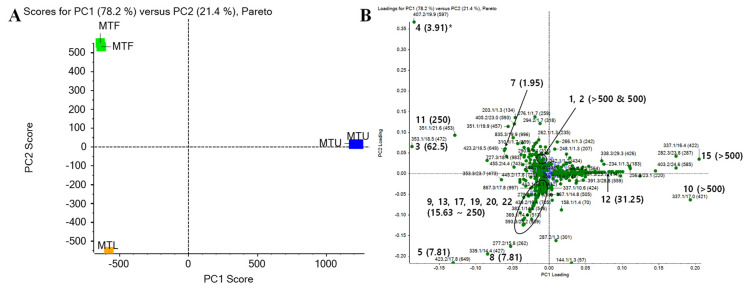
PCA results of extracts of *M. tricuspidata* leaves (MTL), ripe fruits (MTF), and unripe fruits (MTU). (**A**) Score plot and (**B**) loading plot. * Indication (number) of the loading plot is expressed by compound number (MIC value, µg/mL).

To better analyze and visualize similarities and differences among different parts for the antibacterial activities of isoflavones, multivariate data analyses were performed using data from LC-Q-TOF MS. PCA was carried out with a relative amount of 1000 peaks in order of the highest impact for *M. tricuspidata* extracts (Figure 2). In further analysis, 22 isolated isoflavones of *M. tricuspidata* extracts (leaves, ripe fruits, unripe fruits) were found in PCA data, and results are shown in Figure 2B. The first principal factor (PC1) explained 78.2% of the variation across samples, whereas the second principal factor (PC2) explained 21.4% of the variance. The cumulative variance contribution of PC1 and PC2 was 99.6%. In the PCA factor score plot (Figure 2A), MTF and MTL were negatively correlated with PC1, whereas MTU was positively correlated with PC1. In addition, MTL was negatively correlated while MTF was positively correlated with PC2 to distinguish these two extracts. In PC1 of the loading plot (Figure. 2B), most of the isolated compounds showed a negative correlation, indicating that compound compositions of MTF and MTL were similar. However, compounds **5** and **8** (MTL extract), compounds **10** and **15** (MTU extract), and compound **4** (MTF extract) exhibited heavier factor loadings, indicating that these compounds had distinctive features from each extract. This result is similar to a previous report showing that flavonoids with a side chain of cyclization between hydroxyl and prenyl groups at the A-ring are predominant in unripe fruits, whereas flavonoids with a linear prenyl side chain are the main components in ripe fruits [11]. As shown in Table 1, compounds **4**, **5**, and **8** had MICs of 3.91 to 7.81 µg/mL against *S. iniae*, while compounds **10** and **15** had MICs > 500 µg/mL. Similar results were observed for MTF and MTL extracts. They showed much higher antibacterial activities against Gram-positive bacteria than the MTU extract (Table 3). These results indicate that each part’s antibacterial activity and the antibacterial activity of isoflavones isolated from each part are highly correlated. Considering that the antibacterial activity of MTF against *S. iniae* was higher than that of MTL or MTU, MTF-containing compound **4** was considered to be of high value as a material. It could be used as a feed additive. Thus, it was selected for further optimization procedures in the present study. In addition, the use of this ripe fruit as an ingredient in dietary supplements and functional foods ingredients is being actively investigated in many fields [11]. These advantages might make it a promising candidate source for commercialization in aquaculture.

### 3.4. Optimization Procedures

#### 3.4.1. Statistical Analyses and Model Fitting of BBD

A common perception of natural product safety and high consumer acceptance with a belief that “natural” equals “safe” is not only false, but also misleading [39]. To maximize the antibacterial efficacy of the extract without causing toxicity to farmed fish, excellent antibacterial activity and low cytotoxicity were selected as important factors for optimization.

The BBD matrix applied both actual and predicted values of antibacterial activity against *S. iniae* DSJ19 and cytotoxicity to FHM cells, as shown in Table S1. When all cases were considered, experimental data were well fitted by second-order polynomial models (R^2^: 99%). In addition, since experimental and predicted responses were very similar to each other, unexplained variance among experimental data was considered irrelevant. According to ANOVA of a regression model, linear and quadratic terms were significant (*p* < 0.05), indicating that the relationship between the response variable and test variables was not simply a linear one (Table S3). All mathematical models obtained were found to be suitable for the analysis of the response surface, since there was no evidence of inadequacy based on the lack-of-fit test (*p* > 0.05).

Analysis of regression coefficients (Tables S3 and S4) showed that all linear terms (X_1_, X_2_, and X_3_) were negative in all evaluated responses, whereas all quadratic terms (X_1_^2^, X_2_^2^, and X_3_^2^) were positive (*p* < 0.05). This meant that antibacterial activity could be enhanced by X_1_, X_2_, and X_3_ and that there was a curvature effect in the model. In addition, both antibacterial activity and cytotoxicity were positive in quadratic terms, meaning that cytotoxicity and antimicrobial activity showed the same pattern. Regarding the antibacterial activity, as the ethanol concentration and extraction temperature increased, the antibacterial activity also increased. Similarly, cytotoxicity increased with increasing ethanol concentration.

Phenolic compounds found in plants, including flavonoids, simple phenols, and phenolic derivatives, are generally water-soluble [40,41]. These phenolic compounds can also be extracted with organic solvents, particularly aqueous ethanol [42]. Although hot water extraction is a commonly used extraction method for functional ingredients in medicinal plants, previous studies have shown that using organic solvents can shorten the extraction time and increase the extraction efficiency and antibacterial activity [27]. In the present study, 6,8-diprenylgenistein (**4**), the active compound, is a hydrophobic molecule having a di-prenyl group. Ethanol could be more efficient than water when it is extracted from MTF [14,19]. Therefore, ethanol as an organic solvent was considered as the most suitable solvent during the extraction of bioactive substances such as antibacterial compounds from MTF. The relationship between dependent and independent variables was further elucidated by constructing a response surface plot. The effects of X_1_ and X_2_ with their interactions on antibacterial activity and cytotoxicity at a fixed level of X_3_ (mid-level) are shown in Figure S2.

Considering statistically significant (*p* < 0.05) coefficients, the following mathematical models were obtained:

Y = 4430.43 − 136.57X_1_ − 1.95X_2_ + 1.05X_1_^2^ (2, antibacterial activity) (2)

Y = 258.55 − 3.32X_1_ + 0.02X_1_^2^ (3, cytotoxicity) (3)

#### 3.4.2. Multiple Responses Optimization of MTF Extracts and Predictive Capacities of Mathematical Models

The calculated optimal concentration of ethanol and extraction temperature for obtaining the maximal antibacterial activity against *S. iniae* and low cytotoxicity of MTF extracts toward FHM cells were 50% and 80 °C, respectively (Figure S2B and Table 4). Regarding the extraction time, there was no significant difference in the extraction yield for each extraction condition. Thus, a central value (7.5 h) was used (Figure S1). Under these conditions, the calculated desirability indices for antibacterial activity and cytotoxicity were 0.85 and 1, respectively (Figure S2B). The composite desirability (D) value was calculated to be 0.92 (Table 4). After the extract was prepared with optimal conditions, verification experiments were performed. OE-MTF showed potent antibacterial activity against *S. iniae* with an MIC of 40 μg/mL (predictive capacity of 108.5%) and a CC_50_ of 153.18 µg/mL (predictive capacity of 108.9%) in the cytotoxicity assay (Table 4). Therefore, a good agreement between the predicted and experimental values was obtained, indicating a satisfactory predictive capacity of the developed mathematical model of BBD.

Interestingly, the MBC (80 µg/mL)/MIC (40 µg/mL) ratio of OE-MTF against *S. iniae* was 2, indicating that its antibacterial action was bactericidal, which is the same as those of 6,8-diprenylgenistein (**4**) and isoerysenegalensein E (**7**), the most active compounds in MTF (Table 2 and Figure 2). Although isoerysenegalensein E (**7**) also exhibited excellent antibacterial activity, it was confirmed that isoerysenegalensein E (**7**) was present at trace levels in MTF extract through comparison with a standard compound using LC-Q-TOF MS (Figure S4). Therefore, these results suggested that the antibacterial activity of OE-MTF may be due to the active major compound 6,8-diprenylgenistein (**4**) or its synergistic effect with other active compounds.

### 3.5. Correlations among 6,8-Diprenylgenistein (**4**) Content, Antibacterial Activity, and Cytotoxicity According to Extraction Conditions Based on BBD

**Figure 3:**
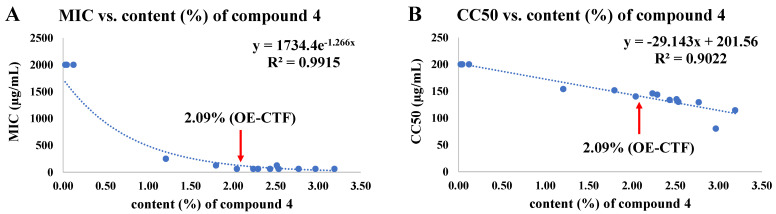
Correlations of content (%) of 6,8-diprenylgenistein (**4**) with antibacterial activity against *S. iniae* (**A**) and cytotoxicity on FHM cells (**B**) according to 15 extracts from BBD.

The active compound, 6,8-diprenylgenistein (**4**), in 15 extracts from BBD was quantified using the quantification method established through method validation (see Supplementary Materials: 2. Quantification of 6,8-diprenlygenistein (**4**) in OE-MTF). The study of the correlations between the content (%) of 6,8-diprenylgenistein (**4**) and the antibacterial activity against *S. iniae* (Figure 3A) and cytotoxicity to FHM cells (Figure 3B) showed that both antibacterial activity and cytotoxicity were highly proportional to the content of 6,8-diprenylgenistein (**4**), with R^2^ values of 0.99 and 0.90, respectively. When the content of 6,8-diprenylgenistein was 2% or more, the MIC value remained almost constant, whereas the cytotoxicity increased as the content increased. Therefore, the optimized extraction conditions obtained through BBD resulted in a meaningful outcome that could satisfy both antibacterial activity and cytotoxicity (the content of 6,8-diprenlygenistein (**4**) was 2.09% in OE-MTF). These results suggest that the antibacterial activity and cytotoxicity of MTF extract are mainly due to the content of 6,8-diprenylgenistein (**4**). Taken together, these results indicate that the 6,8-diprenylgenistein (**4**) content of MTF extract can be used for quality control of products.

### 3.6. Effects of OE-MTF on the Growth and the Morphology of S. iniae

**Figure 4:**
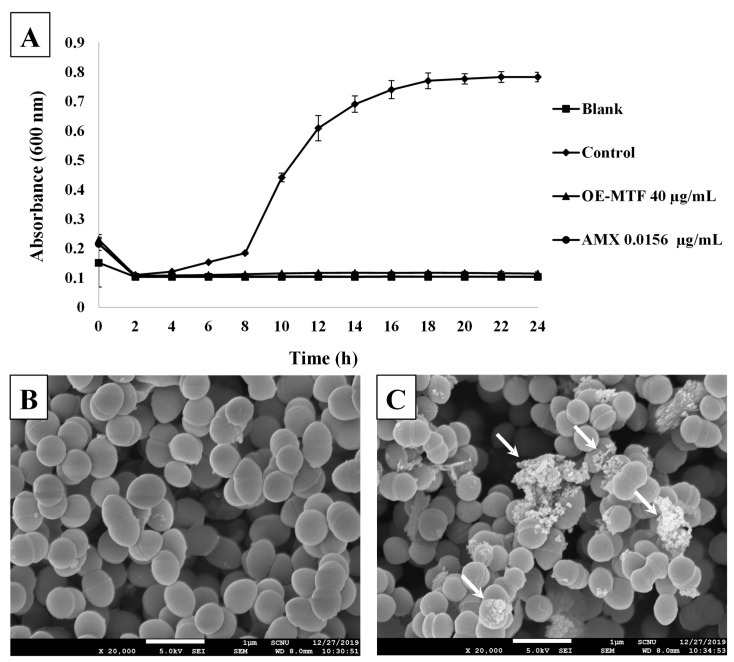
Time-growth curve of *S. iniae* at an MIC of 40 µg/mL (**A**) and scanning electron microscope (SEM) observation of *S. iniae* (**B**,**C**). Morphology of untreated bacteria (**B**, ×20,000) and bacteria treated with OE-MTF at 40 µg/mL for 20 h (**C**, ×20,000). White arrows indicate destruction of bacterial cells with lysed and indistinguishable cytoplasmic membrane structures.

To further confirm the antibacterial activity of OE-MTF against *S. iniae*, a time-growth curve of bacteria was plotted at an MIC of 40 µg/mL. As shown in Figure 4A, OE-MTF strongly inhibited the growth of *S. iniae* at 40 µg/mL, showing the same degree of inhibition as that of amoxicillin. To investigate the effects of OE-MTF on the morphology of *S. iniae*, SEM analysis was performed. As a result, untreated bacteria (Figure 4B) displayed the regular cocci morphology of *S. iniae*. In contrast, OE-MTF induced distinct changes in the morphology of *S. iniae*, such as a lysed and indistinguishable cytoplasmic membrane structure (Figure 4C). These results suggested that OE-MTF disrupted the cytoplasmic membrane of *S. iniae*, leading to their death and disintegration, and could act as a bactericidal agent against *S. iniae*. This bactericidal property of OE-MTF is probably due to bactericidal prenylated isoflavones such as 6,8-diprenylgenistein (**4**). This was confirmed through the correlation study, which exhibited that antibacterial activities of MTF extracts depended on the content of 6,8-diprenylgenistein (**4**). Similarly, Araya-Cloutier et al. [18]. Reported that extracts rich in prenylated isoflavonoids showed potent antibacterial and bactericidal activities against Gram-positive bacteria such as *L. monocytogenes* and MRSA.

## 4. Conclusions

As a result of SAR analysis of 22 isoflavones from *M. tricuspidata* extracts (leaves, ripe fruits, and unripe fruits), it was confirmed that the prenyl group of the isoflavone skeleton was an important key for antibacterial activity against *S. iniae*. Some characteristic prenylated isoflavones containing 6,8-diprenylgenistein (**4**) (MTF), 6,8-diprenylorobol (**5**) (MTL), and alpinumisoflavone (**10**) (MTU) were identified as compounds that largely determined the antibacterial activity of the extract of *M. tricuspidata* from each part through PCA analysis. In addition, the content of 6,8-diprenlygenistein (**4**) in extracts of *M. tricuspidata* ripe fruits showed a positive correlation with antibacterial activity and cytotoxicity to FHM cells. The optimal extraction conditions of *M. tricuspidata* ripe fruits for antibacterial activity and cytotoxicity were successfully obtained using BBD. Based on the results of BBD, the optimal extraction conditions were an ethanol concentration of 50%, an extraction temperature of 80 °C, and an extraction time of 7.5 h. Furthermore, a good agreement between predicted and experimental responses showed a satisfactory predictive capacity of mathematical models developed. Under these optimized conditions, OE-MTF showed potent antibacterial activity, with an MBC/MIC ratio of 2, suggesting a bactericidal action. In addition, OE-MTF exhibited the disintegration of bacterial cytoplasmic membranes and cell disruption against *S. iniae* in the SEM analysis. Taken together, the results of the present study may provide a basic clue that the extract of *M. tricuspidata* ripe fruits and its active compounds can be applied to control Gram-positive fish pathogens. However, further in vivo efficacy and toxicity studies are needed to better understand the therapeutic efficacy and mechanism of action of OE-MTF and its active compounds in farmed fish, which could ultimately lead to their application as eco-friendly antibacterial agents against streptococcosis and aid food safety by reducing the use of antibiotics in the aquaculture industry.

## Figures and Tables

**Table 1:** Antibacterial activities against *S. iniae* of 22 isoflavones from *M. tricuspidata*.

Compounds	Chemical Formula (M.W. ^a^)	Retention Time (min)	Detected Parts from LC-Q-TOF MS ^b^	*S.iniae* KCTC3657
				µg/mL
Genistein (**1**)	C_15_H_10_O_5_ (270.2)	7.73	L, F, U	>500
Orobol (**2**)	C_15_H_10_O_6_ (286.2)	5.78	L, F, U	500
Gancaonin A (**3**)	C_21_H_20_O_5_ (352.4)	18.36	L, F	62.5 ^c^ (500 ^d^)
6,8-Diprenylgenistein (**4**)	C_25_H_26_O_5_ (406.5)	19.83	L, F	3.91 (7.81)
6,8-Diprenylorobol (**5**)	C_25_H_26_O_6_ (422.5)	17.78	L, F, U	7.81 (31.25)
5,7-Dihydroxy-6-(2″-hydroxy-3″-methylbut-3″-enyl)-4′-methoxylisoflavone (**6**)	C_21_H_20_O_6_ (368.4)	-	Not detected	31.25 (125)
Isoerysenegalensein E (**7**)	C_25_H_26_O_6_ (422.5)	18.45	L, F	1.95 (3.91)
Wighteone (**8**)	C_20_H_18_O_5_ (338.4)	14.42	L, F	7.81 (15.63)
Millewanin H (**9**)	C_25_H_26_O_7_ (438.5)	15.92	L	15.63 (62.5)
Alpinumisoflavone (**10**)	C_20_H_16_O_5_ (336.3)	16.96	F, U	>500
4′-*O*-Methylalpinumisoflavone (**11**)	C_21_H_18_O_5_ (350.4)	21.50	L, F, U	250 (>500)
5,3′,4′-Trihydroxy-6″,6″-dimethylpyrano-[2″,3″:7,6]isoflavone (**12**)	C_20_H_16_O_6_ (352.3)	14.67	L, U	31.25 (250)
3′-Hydroxy-4′-*O*-methylalpinumisoflavone (**13**)	C_21_H_18_O_6_ (366.4)	17.44	U	250 (500)
Euchrenone b_8_ (**14**)	C_25_H_24_O_6_ (420.5)	-	Not detected	62.5 (250)
Derrone (**15**)	C_20_H_16_O_5_ (336.3)	16.36	U	>500
5, 3′,4′, 2‴-Tetrahydroxy-2″, 2″-dimethylpyrano-(5″,6″:7,8)-6-(3‴-methyl-3‴-butenyl)isoflavone (**16**)	C_25_H_24_O_7_ (436.5)	-	Not detected	62.5 (250)
4’-*O*-Methylerythrinin C (**17**)	C_21_H_20_O_6_ (368.4)	14.44	L	125 (500)
(±)-1″-*O*-Methylerythrinin F (**18**)	C_21_H_20_O_7_ (384.4)	-	Not detected	250 (>500)
Furowanin A (**19**)	C_25_H_26_O_7_ (438.5)	14.34	L, F	62.5 (250)
4′-*O*-Methyl-2″-hydroxydihydroalpinumisoflavone (**20**)	C_21_H_20_O_6_ (368.4)	15.43	L	125 (500)
Senegalensin (**21**)	C_25_H_26_O_6_ (422.5)	-	Not detected	7.81 (31.25)
Furowanin B (**22**)	C_25_H_26_O_7_ (438.5)	14.87	L, F	31.25 (125)
OTC				0.25 (1)
AMX				0.0078 (0.031)
Cell No. (CFU/mL)				7.2 × 10^5^

All samples were dissolved in DMSO and diluted in broth with the final concentration of DMSO to be 5% (*v*/*v*) or less. ^a^ molecular weight; ^b^ L, F, U = leaves, ripe fruits, and unripe fruits, respectively; ^c^ minimum inhibitory concentration; ^d^ minimum bactericidal concentration; OTC, oxytetracycline; AMX, amoxicillin; CFU, colony forming unit.

**Table 2:** Antibacterial activities of prenylated isoflavones from *M. tricuspidata* against fish pathogenic *Streptococcus* strains.

Compounds	*S. parauberis* KSP44	*S. parauberis* KCTC3651	*S. iniae* DSJ19	*S. iniae* BS9	*S. iniae* KCTC3657
	µg/mL	µg/mL	µg/mL	µg/mL	µg/mL
Genistein (**1**)	250 ^a^ (>250 ^b^)	>500	>500	>500	>500
6,8-Diprenylgenistein (**4**)	1.95 (1.95)	3.91 (15.63)	3.91 (7.81)	3.91 (7.81)	3.91 (7.81)
6,8-Diprenylorobol (**5**)	31.25 (125)	31.25 (250)	7.81 (31.25)	7.81 (31.25)	7.81 (31.25)
Isoerysenegalensein E (**7**)	1.95 (1.95)	1.95 (15.63)	1.95 (3.91)	1.95 (3.91)	1.95 (3.91)
Wighteone (**8**)	15.63 (31.25)	15.63 (62.5)	3.91 (15.63)	3.91 (15.63)	7.81 (15.63)
Senegalensin (**21**)	15.63 (62.5)	62.5 (250)	7.81 (15.63)	7.81 (15.63)	7.81 (31.25)
AMX	0.5 (4)	0.5 (2)	0.0156 (0.031)	0.0078 (0.031)	0.0078 (0.031)
Cell No. (CFU/mL)	5.6 × 10^5^	1.2 × 10^5^	5.5 × 10^5^	4.9 × 10^5^	7.2 × 10^5^

All samples were dissolved in DMSO and diluted in broth with the final concentration of DMSO to be 5% (*v*/*v*) or less. ^a^ minimum inhibitory concentration; ^b^ minimum bactericidal concentration; AMX, amoxicillin; CFU, colony forming unit.

**Table 3:** Antibacterial activities of *M. tricuspidata* extracts against fish pathogenic bacteria.

Sample	*S. iniae* KCTC3657	*S. parauberis* KCTC3651	*E. tarda* KCTC12267	*A. salmonicida* KCCM40239
	µg/mL	µg/mL	µg/mL	µg/mL
MTL	250	1000	2000	1000
MTF	62.5	250	>2000	2000
MTU	>2000	>2000	>2000	2000
OTC	0.25	0.5	0.5	0.125
Cell No. (CFU/mL)	11.6 × 10^5^	11.9 × 10^5^	6.5 × 10^5^	7.7 × 10^5^

All samples were dissolved in DMSO and diluted in broth with the final concentration of DMSO to be 5% (*v*/*v*) or less. OTC, oxytetracycline; CFU, colony forming unit.

**Table 4:** Comparison between actual value and predicted value determined under optimal conditions.

Response	Optimized Condition			Composite Desirability (D)	Actual Values	Predicted Values ^a^	Predictive Capacity (%)
	Ethanol % (X_1_)	Temperature (X_2_)	Time (X_3_)				
Cytotoxicity (CC_50_, µg/mL)	50	80	7.5	0.92	153.18 ± 2.93	140.61	108.9%
Antibacterial activity (µg/mL)					40.00 ± 0.00 ^b^ (80.00 ^c^)	43.40	108.5%

^a^ Predicted value from the mathematical models generated; ^b^ minimum inhibitory concentration; ^c^ minimum bactericidal concentration; CC_50_, 50% cytotoxic concentration.

## Data Availability

The data presented in this study are available on request from the corresponding author.

## Supplementary Materials

The following are available online. Design matrix in the Box–Behnken model (Table S1); regression coefficient estimates and their significance test for the quadratic polynomial model of antibacterial activity and cytotoxicity (Tables S3 and S4); additional experimental details for extraction and isolation: 1. Extraction and isolation of isoflavones from *M. tricuspidata*; additional method validation details for quantification: 2. Quantification of 6,8-diprenlygenistein (**4**) in OE-MTF; analytical conditions for 6,8-diprenylgenistein (**4**) (Table S2); intra- and inter-day precision for method validation (Table S5); accuracy data for 6,8-diprenylgenistein (**4**) in OE-MTF (Table S6); contents of 6,8-diprenylgenistein (**4**) in OE-MTF (Table S7); extraction yields (%) for 15 extraction conditions by BBD (Figure S1); response surface and multiple optimization plot showing effects of mutual interactions between two independent variables (Figure S2); comparison of MRM data between standard mixture and OE-MTF (Figure S3); relative comparison of peaks intensity for 6,8-diprenylgenistein (**4**) and isoerysenegalensein E (**7**) in MTF extract (Figure S4).

## Sample Availability

Some of samples are available from the authors.

## List of Abbreviations

ANOVA	Analysis of variance
BBD	Box–Behnken design
BHIA	Brain heart infusion agar
BHIB	Brain heart infusion broth
CC50	50% cytotoxic concentration
DCM	Dichloromethane
DMSO	Dimethyl sulfoxide
EtOAc	Ethyl acetate
FBS	Fetal bovine serum
FHM	Fathead minnow cells
^1^H-NMR	^1^H-nuclear magnetic resonance
HPLC	High-performance liquid chromatography
IDA	Information-dependent acquisition
LC-Q-TOF MS	Liquid chromatography-quadrupole-time of flight mass spectrometry
LOD	Limit of detection
LOQ	Limit of quantification
MBC	Minimum bactericidal concentration
MeOH	Methanol
MIC	Minimum inhibitory concentration
MRM	Multiple reaction monitoring
MTF	Fresh ripe fruits of *M. tricuspidata*
MTL	Dried leaves of *M. tricuspidata*
MTU	Fresh unripe fruits of *M. tricuspidata*
n-BuOH	*n*-Butanol
NP-MPLC	Silica gel column chromatography
NR	Neutral red
OE-MTF	Optimized extract from MTF
PBS	Phosphate-buffered saline
PCA	Principal component analysis
RP-MPLC	Reverse phase column chromatography
RSD	Relative standard deviation
SAR	Structure–activity relationships
SEM	Scanning electron microscope
UV	Ultraviolet
