# Supplementary material for: Antibacterial Activities of Prenylated Isoflavones from Maclura tricuspidata against Fish Pathogenic Streptococcus: Their Structure-Activity Relationships and Extraction Optimization
Source: Molecules. 2021 Dec 9;26(24):7451. doi: 10.3390/molecules26247451 (PMC8704674; doi:10.3390/molecules26247451)
Supplement: Supplementary file 1 [file molecules-26-07451-s001.zip › molecules-1484747-supplementary.pdf]

Supporting Information

Antibacterial activities of prenylated isoflavones from *Maclura tricuspidata* against fish pathogenic *Streptococcus*: their structure-activity relationships and extraction optimization

Jae-Woong Lim[a], Yang Hee Jo[b], Ji-Seok Choi[a], Mi Kyeong Lee[b], Ki Yong Lee[c] and So Young Kang[a,*]

[a]Department of Aqualife Medicine, Chonnam National University, Yeosu 59626, Republic of Korea

[b]College of Pharmacy, Chungbuk National University, Cheongju 28160, Republic of Korea

[c]College of Pharmacy, Korea University, Sejong 30019, Republic of Korea

[*]To whom correspondence should be addressed;

Postal address: Department of Aqualife Medicine, Chonnam National University, Yeosu 59626, Republic of Korea

E-mail address: sykang1@chonnam.ac.kr

Tel.: +82-61-659-7176; Fax: +82-61-659-7176

1. Extraction, isolation, and identification of isoflavones from *M. tricuspidata*

- General experimental procedures

A Jasco UV-550 spectrometer (Tokyo, Japan) was used respectively, for the measurement of ultraviolet (UV) spectra. ^{1}H-nuclear magnetic resonance (^{1}H-NMR) spectra were recorded on AVANCE 400 or 500 MHz spectrometer (Bruker, Billerica, MA, USA) using $CDCl_3$ and CD_3OD as solvents. ESI-MS data was obtained on LCQ Fleet ion trap MS (Thermo scientific, San Jose, CA, USA). Semi-preparative HPLC was performed using a Waters 515 HPLC pump with a 996 photodiode array detector (Waters, Milford, MA, USA), and Waters Empower software using a Gemini-NX ODS-column (150 × 10.0 mm and 150 × 21.2 mm, Phenomenex, Torrance, CA, USA). Column chromatography procedures were performed using silica gel (200–400 mesh, Fisher Scientific, Cleveland, OH, USA) and Sephadex LH-20 (25–100 μm, Pharmacia Fine Chemical Industries Co., Uppsala, Sweden). Thin-layer chromatography (TLC) was performed using aluminum plates precoated with Kieselgel 60 F_{254} (0.25 mm, Merck, Darmstadt, Germany). After spraying with a color reagent (10% vanillin-H_2SO_4 and 10% H_2SO_4 in EtOH), heating revealed the spots.

Dried leaves of *M. tricuspidata* (0.8 kg) were extracted with 100% MeOH twice, which yielded a methanolic extract (102.4 g). This methanolic extract was then suspended in H_2O and partitioned successively with *n*-hexane (12.2 g), CH_2Cl_2 (15.2 g), EtOAc (4.7 g), and *n*-BuOH (17.7 g).

The CH_2Cl_2 fraction (15.2 g) was subjected to silica gel column chromatography with a mixture of CH_2Cl_2 and MeOH with increasing polarity to give ten fractions (MTLM1 - MTLM10). Fraction MTLM2 was subjected to Sephadex LH-20 with a mixture of CH_2Cl_2 and MeOH (1:1) to give four subfractions (MTLM2A - MTLM2D). Subfraction MTLM2B was purified by semi-preparative HPLC and eluted with AcCN:H_2O (70:30) to give compounds **3** and **11**. Fraction MTLM3 was subjected to column chromatography over Sephadex LH-20 and eluted

with CH_2Cl_2:MeOH (1:1) to yield 3 subfractions (MTLM3A - MTLM3C). Subfraction MTLM3B was purified by semi-preparative HPLC and eluted with AcCN:H_2O (70:30) to give compound **6**. Fraction MTLM4 was subjected to column chromatography over Sephadex LH-20 and eluted with CH_2Cl_2:MeOH (1:1) to yield five subfractions (MTLM4A - MTLM4E). Subfraction MTLM4B was subjected to column chromatography over Sephadex LH-20 and eluted with *n*-hexane:CH_2Cl_2:MeOH (10:10:1) to yield eight subfractions (MTLM4B1 - MTLM4B8). Subfraction MTLM4B3 was purified by semi-preparative HPLC and eluted with AcCN:H_2O (45:55) to give compound **17**. Subfraction MTLM4B6 was purified by semi-preparative HPLC and eluted with AcCN:H_2O (60:40) to give compound **20**. Fraction MTLM4D was subjected to column chromatography over Sephadex LH-20 and eluted with *n*-hexane:CH_2Cl_2:MeOH (10:10:1) to yield 12 subfractions (MTLM4D1 - MTLM4D12). Subfraction MTLM4D1 was purified by semi-preparative HPLC and eluted with AcCN:H_2O (60:40) to give compounds **10** and **15**. Subfraction MTLM4D2 was purified by semi-preparative HPLC and eluted with AcCN:H_2O (60:40) to give compounds **4** and **8**. Compound **21** was obtained from MTLM4D4 by semi-preparative HPLC followed by elution with AcCN:H_2O (60:40). Subfraction MTLM4D7 was purified by semi-preparative HPLC and eluted with AcCN:H_2O (70:30) to give compounds **5**. Subfraction MTLM4D9 was purified by semi-preparative HPLC and eluted with AcCN:H_2O (60:40) to give compound **18**. Subfraction MTLM4D10 was purified by semi-preparative HPLC and eluted with AcCN:H_2O (55:45) to give compounds **7**, **19**, and **22**. Subfraction MTLM4D11 was purified by semi-preparative HPLC and eluted with AcCN:H_2O (55:45) to give compound **9**.

The EtOAc fraction (4.7 g) was subjected to silica gel column chromatography with a mixture of CH_2Cl_2 and MeOH with increasing polarity to give nine fractions (MTLE1 - MTLE9). Fraction MTLE3 was subjected to column chromatography over Sephadex LH-20 and eluted with CH_2Cl_2-MeOH (1:1) to yield six subfractions (MTLE3A - MTLE3F). Subfraction

MTLE3D was purified by semi-preparative HPLC and eluted with AcCN-H_2O (25:75) to give compound **1**. Fraction MTLE5 was subjected to column chromatography over Sephadex LH-20 and eluted with CH_2Cl_2:MeOH (1:1) to yield six subfractions (MTLE5A - MTLE5F). Subfraction MTLE5F was purified by semi-preparative HPLC and eluted with AcCN:H_2O (30:70) to give compound **2**.

Fresh unripe fruits of *M. tricuspidata* (2.8 kg) were extracted successively with 75% ethanol at room temperature. The ethanolic extract (508.2 g) was then suspended in H_2O and partitioned successively with *n*-hexane (30.1 g), CH_2Cl_2 (44.6 g), EtOAc (7.5 g), and *n*-BuOH (35.8 g).

The CH_2Cl_2 fraction (44.6 g) was subjected to silica gel column chromatography followed by gradient elution with *n*-hexane:EtOAc (50:1 ~ 0:100) to obtain 11 fractions (MTUM1 - MTUM11). Fraction MTUM1 was subjected to defatting with a mixture of CH_2Cl_2:MeOH (1:1, add 0.1% H_2O) to give compound **11**. Fraction MTUM6 was subjected to MPLC using CH_2Cl_2:MeOH step-gradient elution (50:1 ~ 0:100) to give 11 subfractions (MTUM6A - MTUM6K). Subfraction MTUM6D was purified by semi-preparative HPLC and eluted with AcCN:H_2O (57:43) to give compound **13**. Compound **10** was obtained by recrystallization of fraction MTUM7. Fraction MTUM7 was subjected to column chromatography over Sephadex LH-20 and eluted with 100% MeOH to yield four subfractions (MTUMC7A - MTUM7D). Subfraction MTUM7C was purified by semi-preparative HPLC and eluted with AcCN:H_2O (55:45) to give compounds **6** and **14**. Subfraction MTUM7D was purified by semi-preparative HPLC and eluted with AcCN:H_2O (57:43) to give compound **3**. Fraction MTUM8 was subjected to column chromatography over Sephadex LH-20 and eluted with 100% MeOH to yield four subfractions (MTUM8A - MTUM8D). Subfraction MTUFM8C was purified by semi-preparative HPLC and eluted with AcCN:H_2O (55:45) to give compounds **15** and **20**. Fraction MTUM9 was subjected to column chromatography over Sephadex LH-20 and eluted

with 100% MeOH to yield four subfractions (MTUM9A - MTUM9D). Subfraction MTUM9B was subjected to column chromatography over Sephadex LH-20 and eluted with CH_2Cl_2:MeOH (1:1) to yield two subfractions (MTUM9B1 - MTUM9B2). Subfraction MTUM9B1 was purified by semi-preparative HPLC and eluted with AcCN:H_2O (57:43) to give compound **17**. Subfraction MTUM9B2 was purified by semi-preparative HPLC and eluted with AcCN:H_2O (57:43) to give compound **7**. Subfraction MTUM9D was purified by semi-preparative HPLC and eluted with AcCN:H_2O (45:55) to give compounds **12** and **16**. Fraction MTUM10 was subjected to column chromatography over Sephadex LH-20 and eluted with 100% MeOH to yield three subfractions (MTUM10A - MTUM10C). Subfraction MTUM10C was purified by semi-preparative HPLC and eluted with AcCN:H_2O (57:43) to give compound **5**. Fraction MTUM11 was subjected to column chromatography over Sephadex LH-20 and eluted with 100% MeOH to yield three subfractions (MTUM11A - MTUM11C). Subfraction MTUM11C was purified by semi-preparative HPLC and eluted with AcCN:H_2O (50:50) to give compound **2**.

The EtOAc fraction (7.5 g) was subjected on silica gel column chromatography followed by gradient elution with CH_2Cl_2:MeOH (50:1 ~ 5:1) to obtain eight fractions (MTUE1 - MTUE8). Fraction MTUE1 was subjected to column chromatography over Sephadex LH-20 and eluted with CH_2Cl_2:MeOH (1:1) to yield five subfractions (MTUE1A - MTUE1E). Subfraction MTUE1E was purified by semi-preparative HPLC and eluted with AcCN:H_2O (30:70) to give compound **1**.

Fresh ripe fruits of *M. tricuspidata* (1.2 kg) were extracted twice with 100 % MeOH, which yielded a methanolic extract (486.5 g). The methanolic extract was suspended in H_2O and partitioned successively with *n*-hexane (8.8 g), CH_2Cl_2 (14.4 g), EtOAc (4.3 g), and *n*-BuOH (19.5 g).

The CH_2Cl_2 fraction (14.4 g) was subjected to MPLC over silica gel and gradient eluted with

n-hexane:EtOAc (20:1 ~ 0:100) and EtOAc:MeOH (100:0 ~ 0:100) to give 11 subfractions (MTFM1 - MTFM11). Fraction MTFM4 was subjected to RP-MPLC over silica gel and gradient eluted with MeOH:H_2O (10:1 ~ 100:0) to give three subfractions (MTFM4A - MTFM4C). Compound **11** was purified from MTFM4B by Sephadex LH-20 using 100% MeOH. Compound **3** was purified from MTFM7 by recrystallization using *n*-hexane:CH_2Cl_2 (1:1). Compound **4** was obtained by recrystallization of MTFM8 using *n*-hexane:CH_2Cl_2 (1:1). Fraction MTFM10 was subjected to Sephadex LH-20 and eluted with CH_2Cl_2:MeOH (1:1) to yield five subfractions (MTFM10A - MTFM10E). Compounds **5** and **8** were obtained from MTFM10D by semi-preparative HPLC followed by elution with AcCN:H_2O (80:20). Fraction MTFM11 was chromatographed over Sephadex LH-20 with CH_2Cl_2:MeOH (1:1) to give Compound **1**.

The EtOAc fraction (4.3 g) was subjected to RP-MPLC and gradient eluted with MeOH:H_2O (1:1 ~ 100:0) to yield five subfractions (MTFE1 - MTFE5). Subfraction MTFE1 was rechromatographed on Sephadex LH-20 using MeOH to afford five subfractions (MTFE1A - MTFE1E). Compound **2** was purified from MTFE1E by recrystallization using MeOH.

Table S1. Design matrix in the Box-Behnken model.

Trial No.	Ethanol % (X_1)	Extraction temperature (X_2)	Extraction time (X_3)	MIC for *S. iniae* DSJ19 (µg/mL, Y_1)		CC_{50} for FHM cells (µg/mL, Y_2)	
	Independent variables			Dependent variables			
				Actual value	Predicted value[a]	Actual value	Predicted value
1	80 (+1)	60 (0)	5 (-1)	62.5	86.0	114.5	120.5
2	80 (+1)	80 (+1)	7.5 (0)	62.5	31.3	140.2	123.8
3	20 (-1)	80 (+1)	7.5 (0)	2000	1984.4	200.0	203.7
4	50 (0)	80 (+1)	5 (-1)	62.5	70.3	129.6	140.0
5	20 (-1)	60 (0)	10 (+1)	2000	1976.6	200.0	194.0
6	50 (0)	40 (-1)	10 (+1)	125	117.2	151.6	141.2
7	80 (+1)	40 (-1)	7.5 (0)	125	140.7	135.0	131.3
8	50 (0)	60 (0)	7.5 (0)	62.5	62.5	133.9	141.2
9	20 (-1)	60 (0)	5 (-1)	2000	2007.8	200.0	185.9
10	50 (0)	80 (+1)	10 (+1)	62.5	101.6	130.2	132.5
11	50 (0)	60 (0)	7.5 (0)	62.5	62.5	146.0	141.2
12	50 (0)	40 (-1)	5 (-1)	250	211.0	154.0	151.7
13	80 (+1)	60 (0)	10 (+1)	62.5	54.7	80.4	94.5
14	20 (-1)	40 (-1)	7.5 (0)	2000	2031.3	200.0	216.4
15	50 (0)	60 (0)	7.5 (0)	62.5	62.5	143.7	141.2

MIC, minimum inhibitory concentration; CC_{50}, 50% cytotoxic concentration; [a], Predicted values from the mathematical models generated.

2. Quantification of 6,8-diprenlygenistein (**4**) in OE-MTF

2.1. LC-MS/MS conditions

Optimized conditions were as follows: curtain gas, 25 psi.; ion source gas 1 and gas 2, 50 psi; gas temperature, 400°C; ion spray voltage, 5500 V; declustering potential, 30 V; and flow rate, 1 mL/min (1/5 splitter used). The analytical column was a Kinetex C_{18} (150 × 4.6 mm, 5 µm; Phenomenex CA, USA) connected to a short pre-column. The column was operated at 40°C. The mobile phase consisted of 0.1% formic acid water solution (A) and methanol (B). MRM high resolution mode was used for acquisition for product ions. Table S2 summarizes retention times, specific transitions, and collision energies used for 6, 8-diprenylgenistein (**4**) and solvent condition.

Table S2. Analytical conditions for 6,8-diprenylgenistein.

Compound	Rt (min)	Q1 mass (m/z)	Q3 mass (m/z)	CE (V)	Solvent condition	
					Time (min)	Methanol (B, %)
6,8-diprenylgenistein (**4**)	8.95	407.5	295.0859	30	0	50
			351.1933	15	10	100
			177.0250	60	15	100
					15.1	50
					20	50

Rt, retention time; CE, collision energy.

2.2. Method validation and contents analyses

Linearity, LOD, and LOQ

Calibration curves for LC-MS/MS analyses were linear over concentration range of 5 to 400 ng/mL for 6,8-diprenylgenistein (**4**) (R^2 of 0.9999). Based on calibration curves, LOD and LOQ were calculated to be 2.00 and 6.07 ng/ml, respectively.

Precision

Method reproducibility was evaluated by intra-day (n = 3) and inter-day (n = 9) variability for three replicate analyses of sample solution. RSD was less than 3%, demonstrating a good precision (Table S5).

Accuracy

The recovery of active compounds was assessed by spiking sample with low, medium, and high concentrations of each compound (5, 20, and 40 ppb). Average recoveries ranged from 101.06% to 102.91% (Table S6).

Specificity

All peaks of 6,8-diprenylgenistein in OE-MTF sample were identified by comparing retention time, parent ions, and product ions with standards in MRM spectra. As a result, high specificity was shown (Fig. S2).

Contents analyses of 6,8-diprenylgenistein (4) in OE-MTF

Average contents of 6,8-diprenylgenistein (**4**) in OE-MTF (1, 2.5, and 5 µg/ml) were calculated to be 2.09 ± 0.04% (Table S7).

Table S3. Regression coefficients estimate and their significance test for the quadratic polynomial model (antibacterial activity)

Term	Coefficient	SE Coefficient	*T*	*P*
Constant	62.500	21.35	2.928	0.033*
Linear				
X_1	-960.938	13.07	-73.507	0.000*
X_2	-39.063	13.07	-2.988	0.031*
X_3	-15.625	13.07	-1.195	0.286
Square				
X_1^2	945.313	19.24	49.126	0.000*
X_2^2	39.062	19.24	2.030	0.098
X_3^2	23.437	19.24	1.218	0.278
Interaction				
X_1X_2	-15.625	18.49	-0.845	0.437
X_1X_3	-0.000	18.49	-0.000	1.000
X_2X_3	31.250	18.49	1.690	0.152
R^2 = 99.9% R^2 (adj) = 99.8%				

*, $P < 0.05$; SE, Standard error.

Table S4. Regression coefficients estimate and their significance test for the quadratic polynomial model (cytotoxicity)

Term	Coefficient	SE Coefficient	*T*	*P*
Constant	141.197	9.456	14.932	0.000*
Linear				
X_1	-41.236	5.791	-7.121	0.001*
X_2	-5.075	5.791	-0.876	0.421
X_3	-4.499	5.791	-0.777	0.472
Square				
X_1^2	17.484	8.524	2.051	0.095
X_2^2	10.117	8.524	1.187	0.289
X_3^2	-9.951	8.524	-1.167	0.296
Interaction				
X_1X_2	1.293	8.189	0.158	0.881
X_1X_3	-8.525	8.189	-1.041	0.346
X_2X_3	0.752	8.189	0.092	0.930
R^2 = 92.4% R^2 (adj) = 78.6%				

*, $P < 0.05$; SE, Standard error.

Table S5. Intra- and inter-day precision

OE-MTF (μg/mL)	Precision: RSD† (%)	
	Intra-day (n = 3)	Inter-day (n = 9)
1	1.56	1.47
2.5	0.96	2.03
5	2.09	2.18

†Relative standard deviation.

Table S6. Accuracy data for 6,8-diprenylgenistein (**4**) in OE-MTF

Compounds	OE-MTF				
	Original (ng/ml)	Spiked (ng/ml)	Detected (ng/ml)	Recovery (%)	RSD† (%)
6,8-diprenylgenistein (**4**)	20.96	5	26.39	101.65	1.58
		20	42.15	102.91	2.12
		40	61.61	101.06	0.92

†Relative standard deviation.

Table S7. Contents of 6,8-diprenylgenistein (**4**) in OE-MTF

OE-MTF (μg/mL)	6,8-diprenylgenistein (**4**)		
	Content (%)	SD[a]	RSD[b] (%)
1	2.10	0.03	1.47
2.5	2.11	0.04	2.03
5	2.06	0.04	2.18
Average	2.09	0.04	2.03

[a] Standard deviation.

[b] Relative standard deviation.

Figure Legends

Figure S1. Extraction yields (%) for 15 extraction conditions by BBD.

Figure S2. Response surface (A) and multiple optimization (B) plot showing effects of mutual interactions between two independent variables.

Figure S3. Comparison of MRM data between standard mixture and OE-MTF (transition of 407.5 to 295.0859 was used for quantifier ion, others (407.5 > 351.1933 and 177.0250) were used as qualifier ions).

Figure S4. Relative comparison of peaks intensity for 6,8-diprenylgenistein (**4**) and isoerysenegalensein E (**7**). (A) Extracted ion chromatogram corresponding to the chemical formulas of 6,8-diprenylgenistein (**4**) and isoerysenegalensein E (**7**) from MTF extract (1 mg/mL) using high-resolution LC-Q-TOF MS. (B) Extracted ion chromatogram corresponding to the chemical formulas of 6,8-diprenylgenistein (**4**) and isoerysenegalensein E (**7**) from compound mixture (100 ng/mL) using high-resolution LC-Q-TOF MS.

Figure S1. Extraction yields (%) for 15 extraction conditions by BBD.

EtOH	Temp	Time	Yield%
80	60	5.0	61.14
80	80	7.5	63.05
20	80	7.5	58.58
50	80	5.0	63.64
20	60	10.0	58.36
50	40	10.0	57.46
80	40	7.5	63.55
50	60	7.5	57.07
20	60	5.0	60.26
50	80	10.0	61.13
50	60	7.5	62.33
50	40	5.0	60.52
80	60	10.0	61.11
20	40	7.5	59.72
50	60	7.5	62.26

Figure S2. Response surface (A) and multiple optimization (B) plot showing effects of mutual interactions between two independent variables.

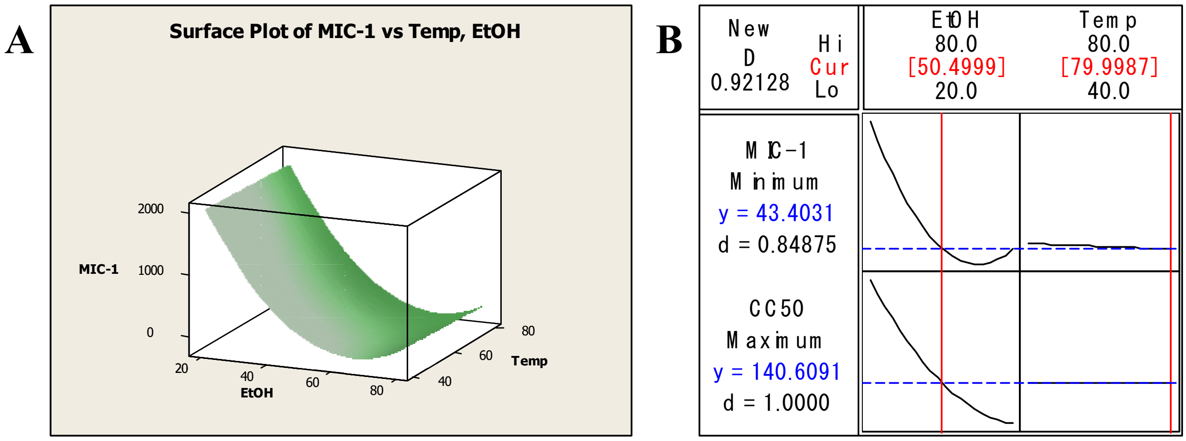

Figure S3. Comparison of MRM data between standard and OE-MTF (transition from 407.5 to 295.0859 was used for quantifier ion; others (407.5 > 351.1933 and 177.0250) were used for qualifier ions).

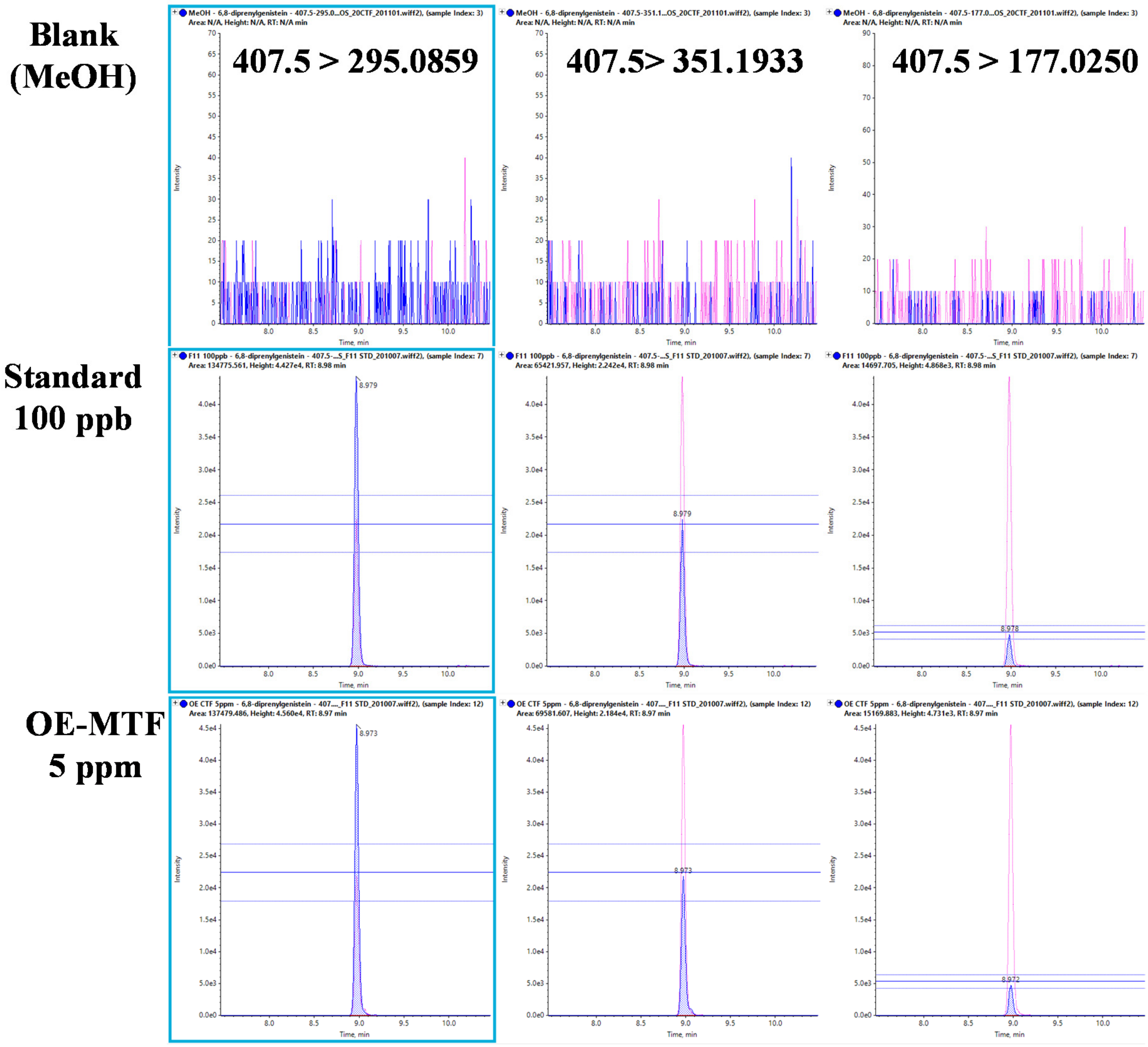

Figure S4. Relative comparison of peaks intensity for 6,8-diprenylgenistein (**4**) and isoerysenegalensein E (**7**). (A) Extracted ion chromatogram corresponding to the chemical formulas of 6,8-diprenylgenistein (**4**) and isoerysenegalensein E (**7**) from MTF extract (1 mg/mL) using high-resolution LC-Q-TOF MS. (B) Extracted ion chromatogram corresponding to the chemical formulas of 6,8-diprenylgenistein (**4**) and isoerysenegalensein E (**7**) from compound mixture (100 ng/mL) using high-resolution LC-Q-TOF MS.

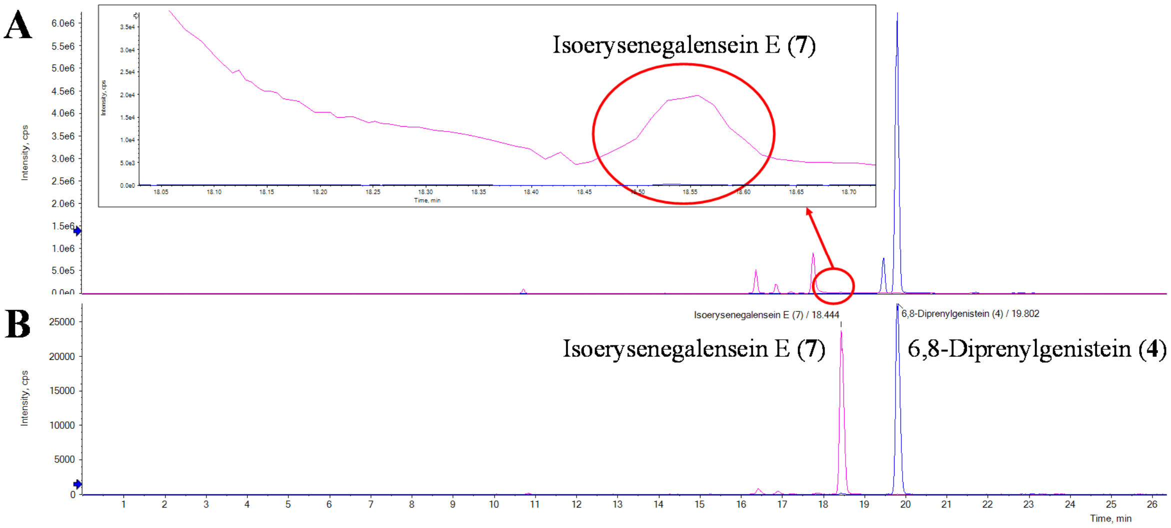

Detailed method for Figure S4

A standard mixture of 100 ng/mL was prepared by mixing 6,8-diprenylgenistein (**4**) and isoerysenegalensein E (**7**), and their chromatographic data were obtained by IDA (Information Dependent Acquisition) mode under the same conditions as Method 2.4 in the manuscript. From the IDA data of MTF extract and compounds, the chromatograms of each compound were extracted for chemical formulas of 6,8-diprenylgenistein (**4**) and isoerysenegalensein E (**7**) by XIC (extracted ion chromatogram) function through the data analysis program, and the peaks of the two compounds in MTF extract and standard mixture were compared.
